# Ecological Responses of Soil Microbial Communities to Heavy Metal Stress in a Coal-Based Industrial Region in China

**DOI:** 10.3390/microorganisms11061392

**Published:** 2023-05-25

**Authors:** Chao Su, Rong Xie, Di Liu, Yong Liu, Ruoyu Liang

**Affiliations:** 1 Institute of Loess Plateau, Shanxi University, Taiyuan 030006, China; suchao@sxu.edu.cn (C.S.);; 2 School of Biosciences, The University of Sheffield, Western Bank, Sheffield S10 2TN, UK; ruoyuliang@hotmail.com

**Keywords:** soil bacterial community, soil fungal community, heavy metal contamination, soil enzyme activity, coal-based industrial activity

## Abstract

Soil microorganisms play vital roles in ecosystem functions, and soil microbial communities might be affected by heavy metal contamination caused by the anthropogenic activities associated with the coal-based industry. This study explored the effects of heavy metal contamination on soil bacterial and fungal communities surrounding different coal-based industrial fields (the coal mining industry, coal preparation industry, coal-based chemical industry, and coal-fired power industry) in Shanxi province, North China. Moreover, soil samples from farmland and parks away from all the industrial plants were collected as references. The results showed that the concentrations of most heavy metals were greater than the local background values, particularly for arsenic (As), lead (Pb), cadmium (Cd), and mercury (Hg). There were significant differences in soil cellulase and alkaline phosphatase activities among sampling fields. The composition, diversity, and abundance of soil microbial communities among all sampling fields were significantly different, particularly for the fungal community. Actinobacteria, Proteobacteria, Chloroflexi, and Acidobacteria were the predominant bacterial phyla, while Ascomycota, Mortierellomycota, and Basidiomycota dominated the studied fungal community in this coal-based industrially intensive region. A redundancy analysis, variance partitioning analysis, and Spearman correlation analysis revealed that the soil microbial community structure was significantly affected by Cd, total carbon, total nitrogen, and alkaline phosphatase activity. This study profiles the basic features of the soil physicochemical properties, the multiple heavy metal concentrations, and the microbial communities in a coal-based industrial region in North China.

## 1. Introduction

Coal has been China’s largest source of energy since the 1950s [1], accounting for about 70% of its primary energy production and consumption [2]. Along with the substantial economic benefits that coal-based industry brings, it also changes the natural environment. Long-term coal mining activities not only cause surface subsidence, land degradation, soil erosion, and vegetation destruction, but also produce a large amount of waste, e.g., coal gangue. Furthermore, coal gangue has been considered one of the most significant sources of soil heavy metal contamination [3,4,5,6]. Additionally, coal combustion activities, such as power generation and steel smelting, also lead to the accumulation of heavy metals in soil [7].

Soil heavy metal contamination has become a severe and widespread problem in China [8]. It has caused broad concern because of the high toxicity, persistence, and nonbiodegradability of heavy metals [9]. Heavy metal contamination not only changes the soil quality but also severely affects the growth and development of crops, posing a significant threat to soil microorganisms, food safety, and human health [10,11]. The soil microbial community structure and enzyme activities are important indices of soil quality. Soil microorganisms are crucial to soil ecosystems due to their roles in regulating the cycling of nutrient elements, maintaining the soil fertility, and improving the quality of plants [12,13]. They drive long-term changes in soil carbon accumulation [14]. It is known that soil microorganisms are sensitive to heavy metal contamination [11,15,16]. For example, soil physicochemical properties and heavy metals significantly affected the structure of the microbial community, particularly for Proteobacteria and Firmicutes [17]. Gołębiewski et al. (2014) found that the diversity and abundance of soil microorganisms near the lead (Pb)–zinc (Zn) mining area had been reduced and that Zn was the largest impact factor [18]. Another study revealed that arsenic (As), Pb, and cadmium (Cd) were the main pollutants surrounding gold tailings, and that heavy metal contamination changed the soil microbial community structure, with Proteobacteria and Actinobacteria being the dominant phyla [19]. Under multiple environmental stresses (e.g., nitrogen loading, phosphorus limitation, and metal contamination), soil microorganisms could alter their functional traits to contribute to soil carbon cycling [20,21]. Therefore, the changes in microbial community structure are often considered to be a sensitive indicator of heavy metal contamination by anthropogenic activities on the soil ecosystems [17,19].

In addition, soil enzymes, acting in biogeochemical cycles of elements, are closely related to the proliferation of soil microbial communities and play a fundamental role in material decomposition [22,23,24]. Decomposition is catalyzed by soil enzymes, which break down dead plants and microbial biomass, and depolymerize macromolecules [14]. On the other hand, soil enzyme activity exhibited high sensitivity to metal-induced stress and was commonly used as a functional indicator of heavy metal contamination [24]. Belyaeva et al. (2005) found that the activities of urease, invertase, catalase, and phosphatase increased under a 24 mg/kg Zn addition treatment [25], while high Zn concentrations in soil significantly decreased the soil enzyme activities and changed the microbial community structure [26,27]. A few studies found that, in the heavy metal contaminated sites near copper smelters, soil enzyme activities were greatly depressed by copper (Cu), Zn, and Pb [28,29]. Cd and mercury (Hg) contamination caused by coal mining also decreased the soil enzyme activities, e.g., catalase, urease, and dehydrogenase [30]. In addition, a nitrogen addition could alter soil enzyme activities, suppressing the activity of lignin-modifying enzymes and enhancing cellulase activity [31]. Changes in enzyme activities could influence microbial carbon use efficiency and subsequently affect soil carbon sequestration in terrestrial ecosystems [14,20].

The bulk of studies have investigated the significant impacts of heavy metals on soil physicochemical properties and microbial communities on coal mining sites [32,33,34,35]. However, it is still unclear how the structure and diversity of soil bacterial and fungal communities change along with different kinds of coal-based industrial activities. Indeed, the dominant pollutants caused by different coal-based activities were a little different. For example, Cd and As were the elements of greatest concern in soils around the coal mining area, followed by Pb, chromium (Cr), and Hg [6]. There was a substantial buildup of As, Cd, Cu, Pb, and Zn in the soils around coal-fired power plants [36], while soils surrounding steel plants were highly polluted by Cd, Zn, Cu, and Pb [37]. Therefore, a deeper insight into the ecological effects of heavy-metal-contaminated agricultural soil surrounding coal-based industrial regions is very essential, and might be useful for land management around enterprise-intensive regions. The main objectives of this study were: (1) to identify the dominant pollutants of each kind of coal-based industrial activity; (2) to determine the effects of different kinds of coal-based activities on soil enzymes and microbial communities; and (3) to explore the relationships between environmental factors and soil microbial (bacteria and fungi) communities.

## 2. Materials and Methods

### 2.1. Study Area and Sample Collection

The study area selected is a developed coal-based industrial region, in Xiangyuan county (112°42′–113°14′ E, 36°23′–36°44′ N), Shanxi province, China. It is estimated that the coal storage capacity of Xiangyuan county is up to 75.8 billion tons, covering an area of about 1160 km^2^. The gross domestic product of Xiangyuan county was ranked second among all counties of Shanxi province due to its coal-based industry. Furthermore, it was selected into the top 100 counties of China with investment potential in 2019. It is located in the North China plain, high in the northwest while low in the southeast, with an average altitude of 1000 m. It has a typical temperate continental climate with annual average temperature and precipitation of 9 °C and 550 mm, respectively. Most soil texture ranges from light loam to clay loam.

Soil samples were collected in November 2019. According to the investigation report of Changzhi city, the coal-based industry in the study area was classified into four kinds, i.e., coal mining industry (CM), coal preparation industry (CP), coal-based chemical industry (CC), and coal-fired power industry (CFP). There are big differences among the number of different industrial plants in the study area. The number of coal mining plants is the biggest, followed by coal preparation plants, coal-fired power plants, and coal-based chemical plants. Therefore, we collected soil samples surrounding industrial plants as much as possible to ensure the accuracy of the results. The sampling was conducted within 1 km of each plant. In addition, samples from farmland (FS) and parks (PS) away from all the industrial plants were collected as references. Except PS samples, all samples of CM, CP, CC, CFP, and FS were collected from agricultural soils vegetated by crops or vegetables. For each sampling site, five soil sampling points (0–20 cm) were well mixed to be homogenized. Thus, a total of 32 soil samples were collected, including 11 CM samples, 6 CP samples, 4 CC samples, 5 CFP samples, 3 FS samples, and 3 PS samples. The collected soil samples were packed into plastic bags and transported to the laboratory with ice packs. Each sample was divided into two parts with one part used for microbial investigation and the other part used for soil properties analysis. For soil physicochemical properties analysis, the air-dried samples were crushed and then passed through 2 mm and 0.149 mm nylon mesh sieves to remove stones and plant roots. The other part was passed through a 2 mm mesh sieve to remove stones and roots first, and then stored at −20 ℃ for DNA extraction and high-throughput sequencing of the soil microbes. 

### 2.2. Soil Physicochemical Determination

The soil pH was measured using a pH meter. The total nitrogen (TN) and total carbon (TC) of the soil were measured using an element analyzer (Vario Macro Cube, Elementar, Hanau, Germany). Nitrate nitrogen (NO_3_^−^–N) and ammonium nitrogen (NH_4_^+^–N) of the soil were measured by the Automated Discrete Analyzer (CleverChem 380, DeChem-Tech, GmbH, Hamburg, Germany). 

The determination of heavy metals, i.e., As, Cd, Cr, Cu, Hg, nickel (Ni), Pb, and Zn, was carried out with reference to Soil environmental quality of China (GB 15628-2018). Firstly, 0.50 g soil samples were weighed, placed into PVC digestion vessels, and then digested using 10 ml mixed-acid of HNO_3_, HClO_4_, HCl, and HF. Each sample weight was between 0.10 and 2.00 g according to the content of the target element. The digestion solution was diluted with 2% HNO_3_ to a final volume of 50 mL. The concentrations of Cr, Zn, Pb, Cu, Ni, and Cd were determined by inductively coupled plasma mass spectrometry (ICP-MS). The concentrations of As and Hg were determined by an atomic fluorescence spectrometer. To ensure the accuracy and precision of the analysis, quality assurance and quality control (QA/QC) procedures were followed by using reagent blanks, analytical duplicates, and standard reference materials in the digestion and determination. The recoveries for metals in the standards were 82–116%.

### 2.3. Soil Enzyme Activities Assays

Four kinds of soil enzymes, i.e., urease (UE), catalase (CAT), cellulase (CL), and alkaline phosphatase (ALP), were studied. Urease, cellulase, and alkaline phosphatase are the most frequently hydrolases, associated with nitrogen and phosphorus acquisitions [38]. Catalase is one of the most frequently assayed oxidases, targeting decomposing substrates [38]. Urease, catalase, cellulase, and alkaline phosphatase were determined by the indophenol blue colorimetry method, potassium permanganate titration method, 3,5-Dinitrosalicylic acid colorimetry method, and phenylenediamine phosphate colorimetry method, respectively [39,40,41]. Each one was determined in 3 replicates in the same treatment and determined according to the steps of the purchased kit (Bioengineering Co., Ltd., (Shanghai, China)).

### 2.4. DNA Extraction, PCR Amplification and Illumina Miseq Sequencing

The DNA of the microbial community was extracted from 0.50 g soil samples using the Fast DNA^®^Extraction Kit (Omega Bio-tek, Norcross, GA, USA). The DNA extract was checked on 1% agarose gel. The DNA concentration and purity were measured by Nanodrop 2000 UV-vis spectrophotometer (Thermo Scientific, Wilmington, DE, USA) [42]. For bacteria, the genomic DNA was amplified by PCR using the 16S rRNA gene V3-V4 region primers with 338F (5′-ACTCCTACGGGAGGCAGCAG-3′) and 806R (5′-GGACTACHVGGGTWTCTAAT-3′) by an ABI GeneAmp^®^ 9700 PCR thermocycler (ABI, CA, USA). For fungi, primers ITS1F (5′-CTTGGTCATTTAGAGGAAGTAA-3′) and ITS2 (5′-GCTGCGTTCTTCATCGATGC-3′) were used. Finally, the sequencing was conducted at Shanghai Majorbio Bio-pharm Technology (Shanghai, China) using the Illumina MiSeq PE300 platform (Illumina, San Diego, CA, USA). 

### 2.5. Analyses of Microbial Community Composition and Divresity

The raw 16S rRNA and ITS gene sequencing reads were quality-filtered by the FASTP (version 0.20.0) and merged by the FLASH (version 1.2.7) with the following criteria: (1) The 300 bp reads were truncated at any site receiving an average quality score of lower than 20 over a 50 bp sliding window, and the truncated reads shorter than 50 bp or containing ambiguous characters were discarded; (2) Only overlapping sequences longer than 10 bp were assembled according to their overlapped sequence, and the maximum mismatch ratio of the overlap region is 0.2. (3) Samples were distinguished according to the barcode and primers, and the sequence direction was adjusted, with exact barcode matching and two nucleotides mismatching in the primer match. 

For both bacteria and fungi, operational taxonomic units (OTUs) with a 97% similarity cutoff were clustered, and chimeric sequences were identified and removed. The taxonomy of each OTU representative sequence was analyzed by Ribosomal Database Project Classifier against the 16S rRNA (Silva 138) and ITS (Unite 8.0) gene database using a confidence threshold of 0.7.

For both bacterial and fungal communities, the relative abundance (%) of individual taxa was estimated by comparing the number of sequences assigned to a specific taxon to the total number of sequences obtained for the selected sample. Alpha diversity (i.e., Shannon, Simpson, Ace, and Chao1) metrics were further conducted based on 97% OTU clusters for each sample. 

### 2.6. Statistical Analyses

Data were expressed as mean ± standard deviation. Basic data processing was conducted by Excel 2016. The assumption of normally distributed data was assessed using the Shapiro-Wilk test, and the homogeneity of variance was evaluated by the Levene test. One-way analysis of variance (ANOVA; Tukey, *p* < 0.05) was used to detect the differences in heavy metal concentrations, physicochemical properties and enzyme activities between groups. The Wilcoxon Rank Sum test (*p* < 0.05) was used to determine the differences in the alpha diversity metrics of microbial communities between groups. All statistical analyses were conducted using SPSS 22.0 and R 4.0.4.

Additionally, we conducted Redundancy Analysis (RDA; *p* < 0.05), Variance Partitioning Analysis (VPA) and Spearman correlation analysis to explain the relationships between soil environmental factors (physicochemical properties, enzyme activities and heavy metals) and microbial community. RDA and VPA were performed by using Canoco 5.0.

## 3. Results 

### 3.1. Soil Physicochemical Properties and Heavy Metal Contamination

There were no significant differences in soil pH, NO_3_^–^–N, NH_4_^+^–N and TN measurements among different industrial fields, while TC contents showed some significant differences (*p* < 0.05; Table 1). All the soil samples were alkaline with pH values greater than 7.50. TC contents in the surrounding soils of the coal preparation industry were higher than samples in the other three industrial fields. Compared to the reference samples, NH_4_^+^–N contents in the farmland samples (9.89 mg/kg) were significantly higher than in other samples. TC and TN in the park samples were much higher than others.

The concentrations of heavy metals (i.e., Cr, Ni, Cu, Zn, As, Pb, Cd, and Hg) in the coal-based industrial area were measured. The concentrations of almost all the elements were greater than the background values of Shanxi province, particularly for As, Pb, Cd, and Hg. However, the concentrations of all elements were much lower than the risk screening values of Soil environmental quality of China (GB 15628-2018). Notably, the mean concentration of As in the study area was closer to the risk screening value. Furthermore, the mean concentration of As in the farmland samples was almost two times higher than the risk screening value, indicating that the agricultural soil ecosystems, crop growth, and agricultural product safety were threatened. This was mostly related to the use of pesticides containing arsenic. Among the different fields, apart from As, Pb, and Hg, there were no significant differences for other elements. For the metal As, the mean concentration of farmland samples was two to three times higher than samples in other fields, but there were no significant differences among the four industrial fields. The mean concentration of Pb in the coal preparation industrial fields was almost significantly four times higher than in other fields, and it was close to one-third of the risk screening values of Soil environmental quality of China (GB 15628-2018). This indicated that there was a great quantity of Pb produced in the process of coal preparation. Additionally, the mean concentration of Hg in the park samples was higher than samples in other fields, which was mostly due to the downtown parks influenced by increasing motor vehicles and the use of sludge fertilizer containing Hg.

**Figure 1:**
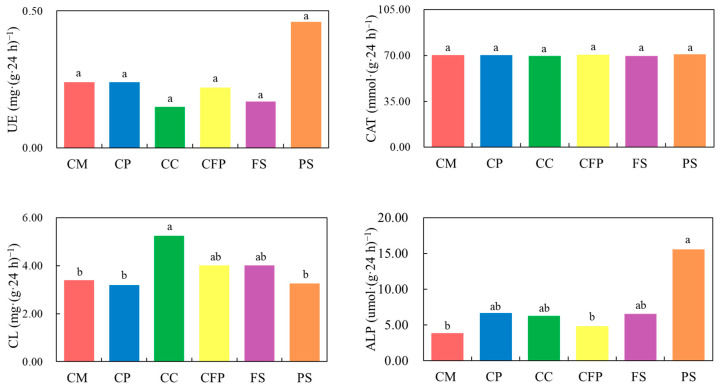
Soil enzyme activities under different sampling fields. Note: UE, urease; CAT, catalase; CL, cellulase; ALP, alkaline phosphatase; CM, coal mining industry; CP, coal preparation industry; CC, coal-based chemical industry; CFP, coal-fired power industry; FS, farmland samples; PS, park samples. Different lowercase letters indicate significant differences (*p* < 0.05, Tukey).

The urease and catalase activities varied between 0.15–0.46 and 68.61–72.85 mg (g·24 h)^−1^ in all samples of the study area, respectively. For these two activities, there were no significant differences among different fields, although the urease activity in the park samples was almost two times higher than in other fields (Figure 1). The cellulase and alkaline phosphatase activities varied between 3.19–5.24 and 3.85–15.53 mg (g·24 h)^−1^, respectively. The cellulase activity in the coal-based chemical industrial fields was significantly higher than in the coal mining, coal preparation, and park samples (Figure 1). The alkaline phosphatase activity in the park samples was significantly higher than in coal mining and coal-fired power industrial fields (Figure 1).

### 3.2. Composition, Abundance and Diversity of Microbial Communities

#### 3.2.1. Bacterial Community Composition and Diversity

For the bacterial community, a total of 1,515,693 quality sequences were obtained from the 32 samples through Illumina MiSeq sequencing of the 16S rRNA genes. A total of 6583 OTUs were identified in this study, and were classified into 36 phyla, 100 classes, 267 orders, 464 families, 931 genera and 1946 species. For the relative abundance of OTUs, Actinobacteria was dominant (36.04%) at the phylum level, followed by Proteobacteria (26.66%), Chloroflexi (12.73%), Acidobacteria (10.49%), Gemmatimonadetes (3.80%), Bacteroidetes (2.53%), Firmicutes (2.01%), Patescibacteria (1.21%), Cyanobacteria (0.96%) and Planctomycetes (0.74%). The 4 dominant phyla (relative abundance > 5%) accounted for 85.92% of the total OTUs and the top 10 phyla made up to 97.17% of all bacterial communities.

**Figure 2:**
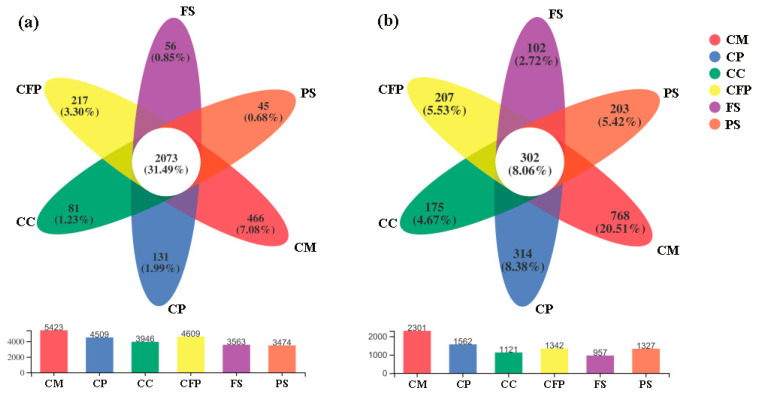
Venn diagram displaying the core and unique OTU numbers across all sampling fields: (**a**) the bacterial community, (**b**) the fungal community. Note: CM, coal mining industry; CP, coal preparation industry; CC, coal-based chemical industry; CFP, coal-fired power industry; FS, farmland samples; PS, park samples.

To explore the adaptation characteristics of bacteria to the local soil habitats in this study area, we analyzed the core and unique OTUs. There were a total of 2073 OTUs (31.49%) shared across all sampling fields (Figure 2a), which were defined as the core microbiome, while the various numbers of OTUs only detected at individual fields were considered to be the unique microbiomes. The predominant members of the 2073 core OTUs belonged to Proteobacteria (25.28%) and Actinobacteria (11.97%) at the phylum level. These core OTUs represent the bacterial communities prevalent in the alkaline heavy metal polluted soil environment surrounding a coal-based industrial region in north China. Last but not least, the total number of OTUs constructed in each sampling field in decreasing order was CM > CFP > CP > CC > FS > PS, as well as the number of unique OTUs (Figure 2a).

**Figure 3:**
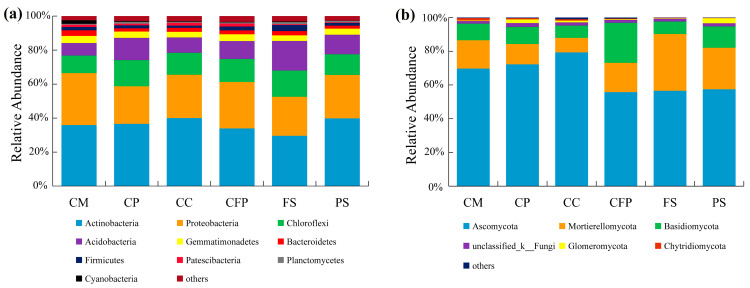
Soil microbial community composition with relative abundance >1% at the phylum level: (**a**) the bacterial community and (**b**) the fungal community. Note: CM, coal mining industry; CP, coal preparation industry; CC, coal-based chemical industry; CFP, coal-fired power industry; FS, farmland samples; PS, park samples.

In addition, slight large but not significant differences in community composition were found across all the sampling fields. Compared with the park samples, the coal-fired power and farmland samples had an obviously lower proportion of Actinobacteria, and the coal preparation samples exhibited a lower proportion of Proteobacteria (Figure 3a). Except for coal mining, coal-based chemical and park samples, the others had a little bigger proportion of Chloroflexi. The farmland and coal preparation samples had a bigger proportion of Acidobacteria than the park samples (Figure 3a).

For bacteria, there were some significant differences in alpha diversity between different sampling fields. Shannon diversity index suggested that samples from the coal mining industrial field possessed lower alpha diversity compared with other sampling fields, while Simpson index showed an opposite result (Table 2). The Ace and Chao1 richness of coal mining samples was the smallest among all sampling fields (Table 2). The park samples presented the highest richness (Ace and Chao1) and a relatively higher alpha diversity, despite their high Hg concentration. 

#### 3.2.2. Fungal Community Composition and Diversity

For the fungal community, 3745 OTUs were identified at 97% sequence similarity, and they were classified into 16 phyla, 53 classes, 131 orders, 303 families, 710 genera and 1292 species. For the relative abundance of OTUs, Ascomycota was dominant (66.85%) at the phylum level, followed by Mortierellomycota (17.29%), Basidiomycota (11.76%), unclassified_k_Fungi (1.83%), Glomeromycota (1.01%), Chytridiomycota (0.84%), Rozellomycota (0.17%), Olpidiomycota (0.14%), Blastocladiomycota (0.04%) and Kickxellomycota (0.03%). The 3 dominant phyla (relative abundance > 5%) accounted for 95.90% of the total OTUs and the top 10 phyla made up 99.96% of all fungal communities.

The number of total OTUs and unique OTUs in each sampling field in decreasing order was CM > CP > CFP > PS > CC > FS (Figure 2b). There were 302 core OTUs (8.06%) shared across all the sampling fields (Figure 2b). The predominant members of the 302 core OTUs belonged to Ascomycota (59.35%) and Mortierellomycota (2.13%) at the phylum level, among which OTU902 (affiliated to genus *Mortierella*) was the most abundant (7.13%).

Additionally, we found somewhat large but not significant differences in community composition across different sampling fields. Almost all the industrial sampling fields (coal-based chemical, coal preparation, and coal mining) had an obviously larger proportion of Ascomycota than the farmland and park samples. However, the farmland and park samples had a much greater proportion of Mortierellomycota than all the industrial sampling fields (Figure 3b). Furthermore, the coal-fired power, park, and coal mining samples had the largest proportion of Basidiomycota, Glomeromycota, and Chytridiomycota, respectively (Figure 3b).

In terms of fungal community diversity, some obvious differences between the industrial sampling fields and reference samples were found. The results showed that both the Ace and Chao1 richness for the four industrial fields and farmland samples were significantly lower than those of the park samples (Table 3). The Shannon diversity index further stressed that, except the PS samples, the other five sampling fields had significantly lower Shannon diversity. Only the Simpson diversity of coal-fired power samples was significantly lower than the coal mining samples (Table 3). These results indicated that the heavy metal contamination was associated with the richness and diversity of the fungal community in the investigated coal-based industrial region.

### 3.3. Relationships between Soil Microbial Communities and Environmental Factors

**Figure 4:**
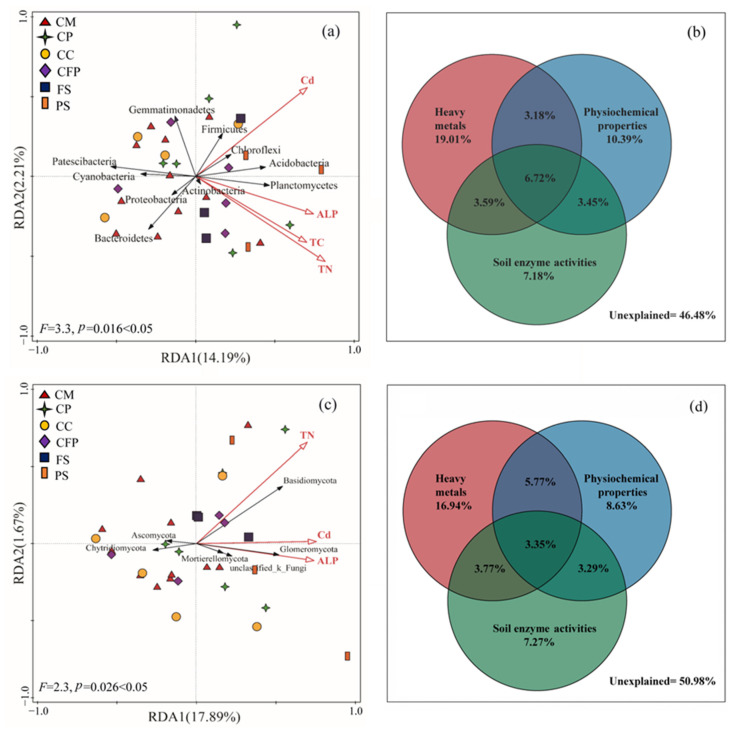
Influences of environmental factors on microbial community abundance at the phylum level: (**a**) RDA for bacteria, (**b**) VPA for bacteria, (**c**) RDA for fungi, and (**d**) VPA for fungi. Note: CM, coal mining industry; CP, coal preparation industry; CC, coal-based chemical industry; CFP, coal-fired power industry; FS, farmland samples; PS, park samples.

For both bacterial and fungal communities, Cd, total nitrogen, and alkaline phosphatase were the significant driving factors on the abundance of the microbial communities (Figure 4a,c). Among the 17 environmental factors, heavy metals explained the biggest proportion of the total variation (Figure 4b,d). Acidobacteria, Chloroflexi, Firmicutest, Planctomycetes, Actinobacteria, Basidiomycota and Glomeromycota had positive correlations with Cd, total nitrogen, and alkaline phosphatase, while Proteobacteria, Bacteroidetes, Cyanobacteria, Gemmatimonadetes, Patescibacteria, Ascomycota and Chytridiomycota had negative correlations with these factors. Additionally, Proteobacteria, Bacteroidetes, Cyanobacteria and Patescibacteria were mainly distributed in the soils surrounding the coal mining industry. Acidobacteria, Chloroflexi, Firmicutest and Planctomycetes were mainly concentrated surrounding the coal preparation industry and park. It corresponded with the information in Figure 3a. Ascomycota was mainly spread in soils surrounding the coal mining industry, coal preparation industry and coal-based chemical industry, which was highly corresponded to the results of Figure 3b. Mortierellomycota was concentrated in soils around farmland and coal mines. The results of the Spearman correlation heatmap corresponded to the RDA, and further revealed the significant correlations between the abundance of predominant microbes and environmental factors (Figures S1 and S2).

## 4. Discussion

### 4.1. Effects of Heavy Metal Contamination on Soil Enzyme Activities

Soil enzymes play a significantly important role in various biochemical processes, particularly in the material cycle and in energy conversion in soil [38,43]. They are rather sensitive to heavy metals, and they are important indicators for evaluating soil contamination [44,45,46]. Urease, widely found in microorganisms and plants, is an essential enzyme in soils that can catalyze the hydrolysis of urea and increase the nitrogen contents in soils [38,47]. In this study, the urease activity in the soil of park samples was almost two times higher than in other fields, consistent with the fact that the TN content of park samples was significantly higher than in most of the other fields. Alkaline phosphatase is a ubiquitous membrane-bound glycoprotein that catalyzes the hydrolysis of phosphate monoesters at basic pH values [48]. The mean alkaline phosphatase activity of park soil was significantly higher than in other fields. This suggested that the concentration of Hg in the park soil was not high enough to inhibit the activities of alkaline phosphatase and urease. 

Catalase is used to catalyze the decomposition of hydrogen peroxide, which is produced in the processes of biological respiration and decomposition of organic matter, to reduce toxicity of hydrogen peroxide to soil organisms [49]. In this study, the heavy metals, especially As, Pb, and Hg had no significant effects on the catalase activity (Table S1). Cellulase is an especially imperative enzyme involved in the decomposition of organic matter into glucose, which is used as the carbon energy source for soil microorganisms [50]. The highest value of cellulase was observed in the coal-based chemical field, significantly higher than those in the coal preparation and park fields, where Pb and Hg concentrations were significantly higher, respectively. This proved the effective inhibition of Pb and Hg on cellulase activity indirectly [50,51]. This inhibition was through either the complexation of Pb and Hg with substrates or through reactions with complex enzyme–substrates and the blocking of the functional groups of cellulase [50]. In particular, the inhibition of Pb on cellulase was presumably due to the interaction blockage of Enzyme–SH groups with Pb or Pb with –COOH groups [50].

### 4.2. Influences of Environmental Factors on Soil Microbial Communities

The influences of long-term heavy metal contamination on microorganisms included changes in the composition, abundance, and diversity of the microbial community. According to the OTUs distribution (Figure 2) and microbial community distribution at the phylum level (Figure 3), we could infer that the six sampling fields in this study had different habitats, and their microbial species were significantly different, especially for the fungal community. Yin et al. (2015) and Zhao et al. (2019) obtained similar findings, and their studies also found that under long-term and high-level heavy metal contamination, the composition, diversity, and abundance of the soil microbial community changed [17,52]. In fact, the sensitive species were more susceptible to long-term heavy metal contamination, while the resistant species would adapt to new habitats and increase their abundance, and hence the microbial community composition altered [17,21]. This might be the reason why a large number of unique OTUs existed in the coal mining, coal preparation, and coal-fired power industrial fields. Notably, the park samples displayed the highest richness (Ace and Chao1) and a relatively higher alpha diversity than other sampling fields, despite their relatively higher Hg concentration (0.09 mg/kg). This might be due to the dual effects of Hg on living microorganisms demonstrated by some studies; high Hg concentrations can change microbial community structure and decrease diversity, while low Hg concentrations stimulate microorganisms and increase diversity [53,54,55]. In the present study, the concentrations of Hg in all the sampling fields were lower (<0.10 mg/kg). This supported the famous intermediate distribution hypothesis.

Generally, Actinobacteria, Proteobacteria, and Acidobacteria were the common predominant bacterial phyla in the heavy-metal-contaminated areas [4,43,55,56]. They could resist the toxicity of heavy metals depending on their complexation and adsorption capacities, and could then adapt to new habitats [11,57,58]. The most abundant phylum in the study area was Actinobacteria, which was consistent with the findings in many other heavy-metal contaminated areas [4,59]. This is most likely because some species affiliated with Actinobacteria (e.g., *Streptomyces*) demonstrate a strong resistance to abiotic stress [60]. Proteobacteria was the second most abundant and widespread phylum, which is known for its resistance to extreme environments and always exists as the predominant bacteria in mining areas [17,61]. Furthermore, Acidobacteria was also usually reported to thrive under severe abiotic stresses such as in high heavy metal concentrations. In short, these existant predominant phyla reflected their adaptation to the tough soil environment in this coal-based industrial region. 

The dominant fungal community in this study was Ascomycota (>50%), Mortierellomycota and Basidiomycota at phylum level, suggesting they were less sensitive to heavy metals. Ascomycota, comprising over 110,000 taxa, was usually reported as the most diverse and abundant fungal phylum in various ecosystems [62,63,64]. In particular, it was also found to be predominant in heavy-metal-contaminated areas or mine-associated ecosystems [37,42,65,66,67]. This was because the complex spores generated from Ascomycota had a superior resistance to metals and other pollutants [68]. Furthermore, the ecological responses of Mortierellomycota and Basidiomycota to the harsh environment were relatively stable [65]. Moreover, Ascomycota, Mortierellomycota, and Basidiomycota were important decomposers in the soil, and played a critical role in the C/N cycling processes [41]. 

In this study, the influences of 17 environmental factors on the soil microbial community structure were identified by RDA, VPA, and Spearman correlation analyses. The results showed that heavy metals were more powerful driving factors in the soil bacterial community structure, as well as the fungal community. The soil physicochemical properties and enzyme activities contributed almost equally. In other words, the formation of the soil microbial community structure depended not only on heavy metals, but also on other environmental factors. This was almost consistent with the results of other studies, which also indicated that the soil microbial community structure was affected by a combination of multiple factors (e.g., toxic heavy metals, pH, and soil nutrients), rather than a single factor [4,27,37,42,66]. 

In particular, Cd, total nitrogen, total carbon, and alkaline phosphatase (Cd > TN > TC > alkaline phosphatase) had significant influences. In this study, the Cd concentration was significantly positively correlated with the abundance of Acidobacteria, Planctomycetes, Basidiomycota, and Glomeromycota. Similarly, Liu et al. (2020) also indicated that Cd concentrations had positive correlations with microbial abundance in farmland soil at various contaminated levels [11]. This might be due to the fact that Cd could accelerate the microbial metabolism process and increase microbial abundance at low concentrations [11,69]. Furthermore, in this study, the concentrations of Cd were not high enough to inhibit the activities of both bacterial and fungal communities. Meanwhile, most microbes could tolerate Cd toxicity in multiple ways, such as through extracellular precipitation, cell wall adsorption, enzymatic oxidation, and intracellular complexation, etc. [11,70]. Moreover, we found that Acidobacteria, Planctomycetes, and Basidiomycota were positively correlated with TN and TC. The soil nutrients exhibited a certain regulatory effect on the toxicity of heavy metals, because they could provide the nutrients for microorganism growth and reproduction [17,42,71,72]. The TC might provide the nutrients that allow the more appropriately adapted microbial communities to grow, dominate, and exclude other members [73,74]. The TN played an important role in constructing the microbial communities due to their vital functions in cellular metabolism processes such as cell division, protein synthesis, etc. [75]. The soil nutrients could also influence microbial communities through their effects on plant diversity, since microorganisms depend on litter and root exudates [73,76]. It should be noted that the present study explored the effects of heavy metal contamination on soil microbial communities in view of composition, diversity, and abundance. Therefore, the specific functional genes and related metabolic pathways of the microbial communities should be considered in the future.

## 5. Conclusions

Microorganisms play important roles in maintaining and regulating ecosystems, yet their composition, diversity, and abundance are driven by various environmental factors. In this study, based on high-throughput sequencing, we provided a comprehensive view into the composition, diversity, and abundance of soil microbial communities (i.e., bacteria and fungi) under the heavy metal stress associated with coal-based industrial activities. Considerable differences in the bacterial and fungal communities at the phylum level were found among different industrial fields. In addition, the microbial communities were significantly affected by a combination of heavy metal (Cd), physicochemical properties (TN, TC), and enzyme activities (alkaline phosphatase), with Cd being the primary driving factor. This might affect microbial functionality and biochemical processes such as carbon, nitrogen, phosphorus, and sulfur cycling in soils, which should be further confirmed. Moreover, the highly abundant bacteria and fungi were found under heavy metal stress in this coal-based industrial area. In the future, how these highly abundant microorganisms respond to the environmental factors should be further explored, so as to provide a better understanding of bioremediation in this heavy-metal-contaminated region. 

## Figures and Tables

**Table 1:** Soil physicochemical properties and heavy metal concentrations in different sampling fields.

	CM (*n* = 11)	CP (*n* = 6)	CC (*n* = 4)	CFP (*n* = 5)	FS (*n* = 3)	PS (*n* = 3)	Study Area	Background Value of Shanxi Province	Risk Screening Values *
pH	8.70 ± 0.14 a	8.72 ± 0.12 a	8.74 ± 0.20 a	8.72 ± 0.08 a	8.65 ± 0.06 a	8.57 ± 0.13 a	8.70 ± 0.13	-	-
NO_3_^–^–N (mg/kg)	39.10 ± 7.75 a	45.42 ± 9.16 a	38.27 ± 1.18 a	37.20 ± 3.54 a	36.42 ± 8.84 a	39.37 ± 11.25 a	39.39 ± 7.56	-	-
NH_4_^+^–N (mg/kg)	3.93 ± 1.56 b	4.86 ± 1.91 b	3.89 ± 2.04 b	4.29 ± 1.71 b	10.10 ± 0.53 a	4.17 ± 0.70 b	4.89 ± 2.41	-	-
TC (g/kg)	19.22 ± 13.46 b	36.46 ± 7.96 a	19.66 ± 2.92 b	23.64 ± 12.32 ab	24.52 ± 1.39 ab	31.49 ± 7.87 a	25.18 ± 13.35	-	-
TN (g/kg)	0.70 ± 0.44 b	1.03 ± 0.62 ab	0.50 ± 0.19 b	0.88 ± 0.48 ab	1.05 ± 0.09 ab	1.36 ± 0.30 a	0.86 ± 0.47	-	-
Cr (mg/kg)	59.92 ± 10.29 a	54.47 ± 6.62 a	57.48 ± 18.68 a	61.41 ± 11.94 a	61.87 ± 3.49 a	65.03 ± 7.16 a	59.33 ± 10.22	55.30	250
Ni (mg/kg)	35.67 ± 6.33 a	34.59 ± 4.32 a	36.50 ± 11.42 a	33.88 ± 6.42 a	33.98 ± 4.27 a	34.96 ± 1.24 a	34.85 ± 6.03	29.90	190
Cu (mg/kg)	26.70 ± 4.17 a	27.85 ± 6.10 a	26.73 ± 7.92 a	26.19 ± 4.66 a	27.60 ± 4.47 a	28.97 ± 2.33 a	26.97 ± 4.80	22.90	100
Zn (mg/kg)	73.93 ± 14.60 a	79.23 ± 16.69 a	69.20 ± 21.53 a	69.52 ± 10.77 a	70.11 ± 6.20 a	75.94 ± 2.81 a	72.91 ± 13.93	63.50	300
As (mg/kg)	23.09 ± 13.45 b	14.14 ± 2.80 b	20.68 ± 6.89 b	24.03 ± 12.46 b	41.08 ± 4.37 a	13.80 ± 1.64 b	22.47 ± 11.89	9.10	25
Pb (mg/kg)	18.95 ± 4.08 b	84.70 ± 151.5 a	19.97 ± 4.80 b	19.75 ± 6.00 b	19.47 ± 3.13 b	24.88 ± 2.58 b	31.61 ± 65.18	14.70	170
Cd (mg/kg)	0.19 ± 0.04 a	0.24 ± 0.06 a	0.18 ± 0.05 a	0.21 ± 0.05 a	0.23 ± 0.06 a	0.24 ± 0.02 a	0.21 ± 0.05	0.102	0.6
Hg (mg/kg)	0.03 ± 0.02 b	0.05 ± 0.02 ab	0.02 ± 0.01 b	0.04 ± 0.03 ab	0.04 ± 0.01 ab	0.09 ± 0.08 a	0.04 ± 0.03	0.023	3.4

Abbreviations: CM, coal mining industry; CP, coal preparation industry; CC, coal-based chemical industry; CFP, coal-fired power industry; FS, farmland samples; PS, park samples; NO_3_^–^–N, nitrate nitrogen; NH_4_^+^–N, ammonium nitrogen; TN, total nitrogen; TC, total carbon; Cr, chromium; Ni, nickel; Cu, copper; Zn, zinc; As, arsenic; Pb, lead; Cd, cadmium; Hg, mercury. * means risk screening values of agricultural land in Soil environmental quality of China (GB 15628-2018). Data are shown as means ± standard deviation. Different lowercase letters in the same row represent a significant difference at 0.05 level (*p* < 0.05, Tukey) between different sampling fields by one-way ANOVA.

**Table 2:** Alpha diversity indices of bacteria in soils in different sampling fields.

Sampling Field	Shannon	Simpson	Ace	Chao1
CM	5.73 b	0.0478 a	2869 b	2615 b
CP	6.34 a	0.0077 c	3038 ab	3017 a
CC	6.01 ab	0.0157 b	3073 ab	2875 ab
CFP	6.51 a	0.0048 c	3224 ab	3194 a
FS	6.52 a	0.0045 c	3190 ab	3235 a
PS	6.44 a	0.0045 c	3418 a	3261 a

Note: Data are shown as median values. Different lowercase letters in the same column represent significant difference at 0.05 level (*p* < 0.05, Wilcoxon Rank Sum test) among different sampling fields. CM, coal mining industry; CP, coal preparation industry; CC, coal-based chemical industry; CFP, coal-fired power industry; FS, farmland samples; PS, park samples.

**Table 3:** Alpha diversity indices of fungi in soils in different sampling fields.

Sampling Field	Shannon	Simpson	Ace	Chao1
CM	3.48 b	0.1359 ab	548 b	549 b
CP	3.45 b	0.1250 ab	574 b	574 b
CC	3.82 ab	0.0886 b	511 b	514 b
CFP	3.19 b	0.1897 a	560 b	568 b
FS	3.58 b	0.0872 b	626 b	631 b
PS	4.10 a	0.0605 b	903 a	900 a

Note: Data are shown as median values. Different lowercase letters in the same column represent significant difference at 0.05 level (*p* < 0.05, Wilcoxon Rank Sum test) among different sampling fields. CM, coal mining industry; CP, coal preparation industry; CC, coal-based chemical industry; CFP, coal-fired power industry; FS, farmland samples; PS, park samples.

## Data Availability

The data presented in this study are available on request from the corresponding author.

## Supplementary Materials

The following supporting information can be downloaded at: https://www.mdpi.com/article/10.3390/microorganisms11061392/s1. Table S1: Pearson correlation coefficients between heavy metals and soil enzyme activities; Figure S1: Spearman correlation heatmap of predominant bacteria and environmental factors; Figure S2: Spearman correlation heatmap of predominant fungi and environmental factors.
