# Supplementary material for: Ecological Responses of Soil Microbial Communities to Heavy Metal Stress in a Coal-Based Industrial Region in China
Source: Microorganisms. 2023 May 25;11(6):1392. doi: 10.3390/microorganisms11061392 (PMC10303547; doi:10.3390/microorganisms11061392)
Supplement: Supplementary file 1 [file microorganisms-11-01392-s001.zip › microorganisms-2365492-supplementary.pdf]

Ecological Responses of Soil Microbial Communities to Heavy Metal Stress in a Coal-Based Industrial Region in China

Chao Su [1], Rong Xie [1], Di Liu [1], Yong Liu [1,*] and Ruoyu Liang [2]

[1] Institute of Loess Plateau, Shanxi University, Taiyuan 030006, China
[2] School of Biosciences, The University of Sheffield, Western Bank, Sheffield S10 2TN, UK
[*] Correspondence: liuyong@sxu.edu.cn; Tel./Fax: +86-0351-7010700

Contents

Table S1. Pearson correlation coefficients between heavy metals and soil enzyme activities.

Figure S1. Spearman correlation heatmap of predominant bacteria and environmental factors.

Figure S2. Spearman correlation heatmap of predominant fungi and environmental factors.

Table S1. Pearson correlation coefficients between heavy metals and soil enzyme activities

	Cr	Ni	Cu	Zn	As	Cd	Pb	Hg	UE	CAT	CL	ALP
Cr	1	0.682**	0.643**	0.637**	0.060	0.417*	0.060	0.090	-0.050	0.037	-0.039	0.123
Ni	0.682**	1	0.827**	0.774**	0.042	0.290	0.157	-0.217	-0.239	-0.070	0.030	-0.187
Cu	0.643**	0.827**	1	0.885**	0.052	0.504**	0.367*	0.056	-0.046	0.143	-0.098	0.076
Zn	0.637**	0.774**	0.885**	1	-0.024	0.609**	0.469**	0.036	0.020	0.112	-0.202	0.011
As	0.060	0.042	0.052	-0.024	1	0.018	-0.096	-0.020	-0.149	-0.307	0.093	-0.182
Cd	0.417*	0.290	0.504**	0.609**	0.018	1	0.469**	0.169	0.169	0.099	-0.185	0.120
Pb	0.060	0.157	0.367*	0.469**	-0.096	0.469**	1	-0.109	-0.092	-0.169	-0.135	-0.087
Hg	0.090	-0.217	0.056	0.036	-0.020	0.169	-0.109	1	0.246	0.322	-0.269	0.706**
UE	-0.050	-0.239	-0.046	0.020	-0.149	0.169	-0.092	0.246	1	0.395*	-0.308	0.296
CAT	0.037	-0.070	0.143	0.112	-0.307	0.099	-0.169	0.322	0.395*	1	-0.268	0.460**
CL	-0.039	0.030	-0.098	-0.202	0.093	-0.185	-0.135	-0.269	-0.308	-0.268	1	-0.009
ALP	0.123	-0.187	0.076	0.011	-0.182	0.120	-0.087	0.706**	0.296	0.460**	-0.009	1

Note: **$p < 0.01$, *$p < 0.05$. UE, urease; CAT, catalase; CL, cellulase; ALP, alkaline phosphatase.

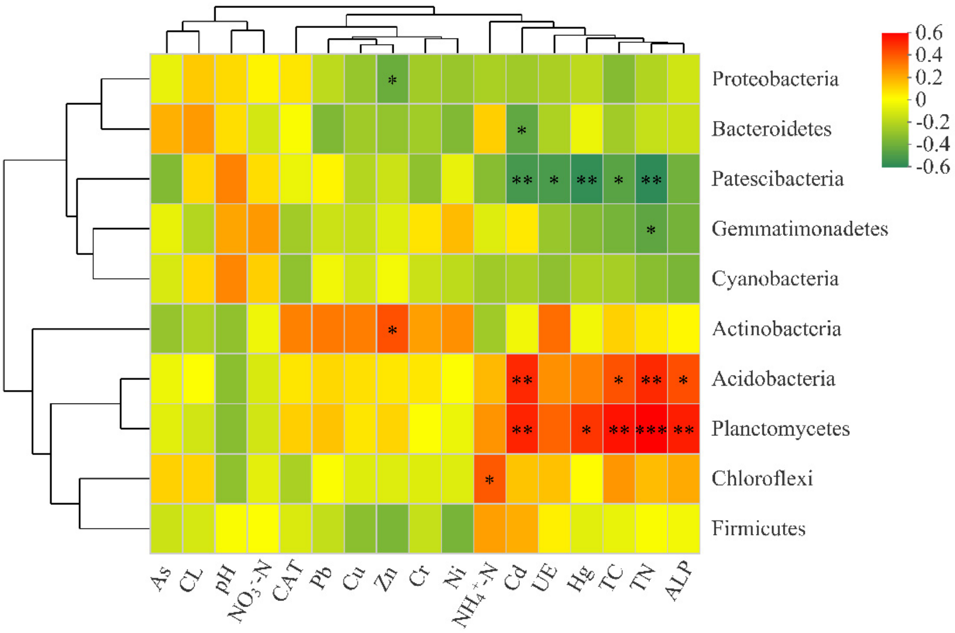

Figure S1. Spearman correlation heatmap of predominant bacteria and environmental factors. Note: ***$p < 0.001$, **$p < 0.01$, *$p < 0.05$.

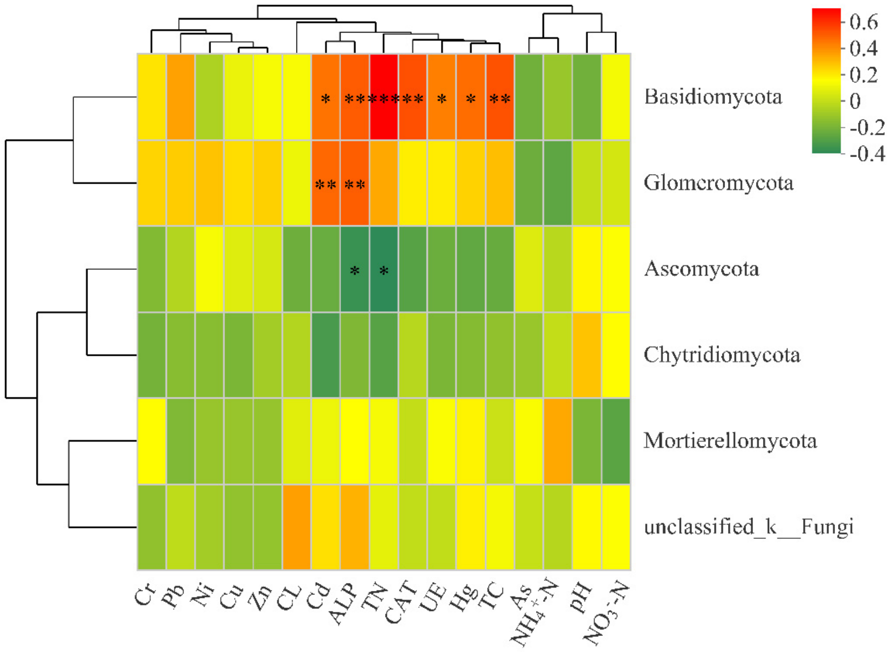

Figure S2. Spearman correlation heatmap of predominant fungi and environmental factors. Note: ***$p < 0.001$, **$p < 0.01$, *$p < 0.05$.
